# Apobec 3G Efficiently Reduces Infectivity of the Human Exogenous Gammaretrovirus XMRV

**DOI:** 10.1371/journal.pone.0011738

**Published:** 2010-07-23

**Authors:** Kristin Stieler, Nicole Fischer

**Affiliations:** Institute for Medical Microbiology, Virology and Hygiene, University Medical Center Eppendorf, Hamburg, Germany; Institut Pasteur Korea, Republic of Korea

## Abstract

**Background:**

The human exogenous gammaretrovirus XMRV is thought to be implicated in prostate cancer and chronic fatigue syndrome. Besides pressing epidemiologic questions, the elucidation of the tissue and cell tropism of the virus, as well as its sensitivity to retroviral restriction factors is of fundamental importance. The Apobec3 (A3) proteins, a family of cytidine deaminases, are one important group of host proteins that control primary infection and efficient viral spread.

**Methodology/Principal Findings:**

Here we demonstrate that XMRV is resistant to human Apobec 3B, 3C and 3F, while being highly susceptible to the human A3G protein, a factor which is known to confer antiviral activity against most retroviruses. We show that XMRV as well as MoMLV virions package Apobec proteins independent of their specific restriction activity. hA3G was found to be a potent inhibitor of XMRV as well as of MoMLV infectivity. In contrast to MoMLV, XMRV infection can also be partially reduced by low concentrations of mA3. Interestingly, established prostate cancer cell lines, which are highly susceptible to XMRV infection, do not or only weakly express hA3G.

**Conclusions:**

Our findings confirm and extend recently published data that show restriction of XMRV infection by hA3G. The results will be of value to explore which cells are infected with XMRV and efficiently support viral spread *in vivo*. Furthermore, the observation that XMRV infection can be reduced by mA3 is of interest with regard to the current natural reservoir of XMRV infection.

## Introduction

XMRV is the first gammaretrovirus identified in a bonafide human infection [1]. The virus is associated with up to 23% of all prostate cancers [2] and was recently found in 67% of PBMCs isolated from patients with chronic fatigue syndrome (CFS) [3]. The frequency of XMRV detection varies from 0–23% in the case of prostate cancer [1], [2], [4]–[6] and 0–67% in PBMCs from CFS patients [3], [7]–[9], putative explanations for the observed discrepancies are geographical localization of the virus or (more likely) detection methods used. Whether or not XMRV infection plays a causative role in the aetiology of these two diseases is presently unclear.

XMRV shares a high degree of sequence identity (∼94% overall, 87% in the gag p62 region, 66% in the env gp75 region) with known endogenous and exogenous murine leukaemia viruses (MLVs) [1]. Like all members of the gammaretrovirus family, it is a simple retrovirus which encodes only *gag*, *pol* and *env*, but no accessory proteins. To date, comprehensive and conclusive epidemiological data about the prevalence of XMRV in the general population, its natural reservoir and route of transmission is lacking. Recently, we have demonstrated that XMRV displays a xenotropic host range in vitro and efficiently infects different cell types as well as cell lines from different species [10]. However, in vivo, the virus so far has been identified only in stromal fibroblasts and epithelial cells in prostate cancer tissue, or in PBMCs from CFS patients [1]–[3]. Additionally, we and others have recently shown that viral restriction can not be fully explained by receptor distribution or LTR-activity, although the latter is significantly increased in cells of prostate tissue origin [10]–[12]. This observation, suggests that other cellular factors, e.g. host restriction factors, are likely to be implicated in the control of viral infection in vivo.

Retroviral restriction factors such as the Apobec 3 family play an important role in species tropism, establishment of a viral infection in vivo and successful spread of the virus. The family of the Apobec3 proteins consists of 7 members (hA3A, hA3B, hA3C; hA3DE; hA3F; hA3G and hA3H) in humans and primates [13]. hA3G displays retroviral activity against a variety of retroviruses (HIV; SIV; HTLV; MLV) [14], LTR retrotransposons and non-LTR retrotransposons [15]–[20]. In addition, hA3G and hA3F interfere with the HBV life cycle in cell lines [21], [22]. In contrast to the various Apobec3 proteins expressed in humans and primates, the mouse genome contains only one gene encoding mA3. Besides of the full length protein, alternative splicing and exon skippping also produce a more broadly expressed, shorter isoform of mA3 (mA3ΔExon5) [23]. The principal ability of the mouse Apobec 3 protein to efficiently restrict virus infection has been demonstrated for the betaretroviruses Mason-Pfizer Monkey Virus (MPMV) [24] and Mouse Mammary Tumor Virus (MMTV) [25] as well as for friend murine leukaemia virus (FrLV) [26]. In contrast, its activity against Moloney murine leukaemia virus (MoMLV) has been reported to be much weaker [23], [27]–[29].

Apobec3 proteins are cytidine deaminases which, when incorporated in viral particles, may enzymatically alter the nascent retroviral DNA via deamination of cytidine to uridine in the DNA minus strand during reverse transcription, hence leading to G-to-A hypermutation. In addition, there are as of yet incompletely understood antiretroviral mechanisms which appear to be independent of the deaminase activity [14].

How the human immunodeficiency virus (HIV) encoded vif protein counteracts hA3G restriction has been the focus of several studies in the past [13], [30], [31]. Vif accelerates proteasomal degradation of the hA3G protein and additionally hinders hA3G mRNA translation [32]. The Retroviruses HTLV or MLV do not encode vif-like proteins and have evolved other strategies to evade Apobec restriction. In the case of HTLV, a peptide motif within the NC (nucleocapsid) region inhibits hA3G encapsidation into the virion [33]. This motif is highly conserved among primate T-cell leukaemia viruses; however, it is not present in other retroviral nucleocapsid sequences [34]. Nevertheless, virion exclusion has been suggested as a common strategy of vif-deficient retroviruses to circumvent Apobec restriction, and exclusion of mA3 from MLV virions has been demonstrated by some groups [35] although not by others [23], [29], [36]. In contrast, hA3G is efficiently packaged in MLV virions, significantly reduces their infectivity and leads to hypermutation of the viral genome [29]. MLV may be protected from mA3 due to the inability of the protein to bind to the MLV gag NC region [35], [37]. Another study suggested that MLV employs two distinct mechanisms; viral RNA blocks mA3 binding to the Gag protein and viral protease cleaves and subsequently inactivates the mA3 protein after maturation of virions [38].

Since human Apobec proteins have been demonstrated to restrict gammaretroviral infection we sought to determine whether XMRV infection can be reduced by the human Apobec proteins A3B, A3C, A3F and A3G. In addition, since XMRV is the first xenotropic MLV identified in a human infection, we were interested in significant differences between MLV and XMRV with regard to sensitivity against human and mouse Apobec 3 proteins. Our results show that XMRV infection is highly restricted only by hA3G, whereas hA3B and hA3F do not significantly reduce XMRV or MLV infection, and that XMRV infection is significantly reduced at low mA3 expression levels, whereas MLV infection is not.

While our manuscript was being prepared and revised, two reports addressing the restriction of XMRV infection by known retroviral restriction factors were published [11], [39]. In the first study, Groom and coworkers comprehensively analyzed XMRV restriction to the major blocks in retroviral life cycle, entry (receptor usage), release of the nucleocapid into the cytoplasm (TRIM5α, Fv1), reverse transcription (Apobec protein family) as well as virion release from the host cell (Tetherin). In the second study, Paprotka et al. investigated inhibition of viral infection as well as the degree of hypermutation imposed upon XMRV genomes by the Apobec3 proteins hA3G, hA3B and mA3. Both studies conclude that XMRV infection can be significantly restricted by the human Apobec 3G protein, whereas the mouse Apobec 3 protein only slightly reduces XMRV infection. In addition, the study by Groom et al. shows that XMRV is restrictive against all TRIM5α proteins tested and highly sensitive against restriction by the mouse Fv1 gene product as well as the human tetherin protein. Furthermore, Paprotka and co-workers could show that hA3G mRNA is nearly undetectable in established prostate cancer epithelial cell lines and XMRV viral genomes from prostate cancer cell lines do not or only rarely contain hypermutations. This, together with the low genetic diversity of XMRV genomes observed so far, suggests that XMRV replication in vivo most likely takes place in cells which not, or only weakly, express hA3G.

Our findings are in overall good agreement with above studies, confirming that XMRV infection is highly restricted only by hA3G [11], [39], whereas hA3B and hA3F do not significantly reduce XMRV or MLV infection. Our results are furthermore supportive of the observation by Groom et al. [11] that XMRV is partially restricted by mA3. We show that XMRV infection, but not infection by MLV, is significantly reduced at low mA3 expression levels.

We have also analyzed mRNA expression levels of hA3B, C, F and G in cell lines as well as primary cells from the prostate and lymphoid compartments, i.e. those cell types which have been reported to support XMRV infection in vivo. Lymphoid cells were found to express high levels of hA3G, whereas (in accord with Paprotka et al. 2010) hA3G expression levels in established prostate cancer cell lines were undetectable. However, primary stromal cell lines from different prostate cancer patients display variable hA3G expression levels which range from the undetectable to levels which are comparable to those found in cells from the haematopoietic compartment.

## Materials and Methods

### Cell culture

The human cell lines TE 671 (ATCC #CCL-136), DU145 (ATCC #HTB-81), 293T (ATCC# CRL-11268), A549 (ATCC# CCL-185), HeLa (ATCC# CCL-2) and 293T cells chronically infected with XMRV (293TX) were cultured in DMEM (Invitrogen) supplemented with 10% FCS and grown at 37°C, 5% CO_2_ and 100% relative humidity. LNCaP (ATCC #CRL-1740), 22Rv1 (ATCC #CRL-2505), Raji (ATCC #CCL-86), Jijoye (ATCC #CCL-87), Reh cells (ATCC #CRL-8286) and U937 cells (ATCC #CRL-1593.2) were grown in RPMI (Invitrogen) supplemented with 10% FCS and L-glutamin (37°C, 5% CO_2_ and 100% relative humidity). Primary stromal fibroblast cell lines were established as described previously [10].

### Plasmids

XMRV env expression plasmid pHCMV-XMRVenv as well as XMRV proviral clone have been described previously [10]. Plasmids used in pseudotyping experiments pSF91-I-eGFP-PRE and pSV-MoMLVGag-pol have been described [40], [41]. pM5-LTR-Luc was cloned by replacing the LacZ reporter gene within the pM5-LacZ-neo plasmid with the firefly luciferase gene. pM5-LacZ-neo was originally constructed by inserting the LacZ gene cassette using the SacII and NotI sites within p5O-M-X-neo [42]. pSV-XMRVgag1940 expression plasmid has been cloned by replacing nt 2612-4946 of the pSV-Mo-MLVgagpol expression plasmid with the corresponding gag sequence of XMRV using XhoI restriction site at position 604 (GI: 88765817) and SacI at position 2544 (GI: 88765817).

Expression plasmids encoding hA3B, hA3C, hA3F and hA3G [43] were obtained by the NIH AIDS Research and Reference Reagent Program. The plasmid expressing the mouse Apobec protein has been described before [44].

### hA3 mRNA expression levels determined by quantitative PCR

Total RNA from cell lines as well as from PBMCs of different patients was isolated using the RNeasy extraction kit (Qiagen, CatNo.74104) according to manufacturer's instructions. 100ng total RNA was DNaseI digested and subjected to RT-PCR with a random hexamer primer and the SuperScript™ Reverse Transcriptase (Invitrogen, CatNo 18064-014). cDNA levels were quantified using a Qiagen Rotorgene Q 5plex instrument and Rotorgene 1.7 software. Reactions were performed in microtubes containing 5µl 2× SyBr Green mastermix (Fermentas), 3.8µl H2O, 0.1µl primer and 1µl cDNA (1∶5 diluted). Reactions were incubated at 95C for 10min, then 40cycles of 95C for 10s, 58C for 40s, then 72C for 15s. The following primer pairs were used: hA3G: 5′-TGGGGGAGATTCTCAGACAC- and 3′- TTCCAAAAGGGAATCACGTC-; hA3B: 5′-GGTCAGCAATTCATGCCTTGGTAC- and 3′-CCCTGTAGATCTGGGCCGGGTCC-; hA3C: 5′-CCCCTCCACCCTGGACCC- and 3′-CGCAGGCTGGAGGAACGGGGTCTGT-; hA3F: 5′-GGCCAGGTGCCCAGGTCTTTC- and 3′-TGCACCAAGACATGAGCTTCCC-. PCR efficiency of each primer set was determined based on standard curves of serial 10fold dilutions of cDNA from Raji or U937 cells. Ct values (determined by using the Rotorgene Software version 1.7) were plotted against the log10 value of template concentration. The slope (M) determines the reaction efficiency according to (10^−1/M^)−1 = 1 (supplementary figure S1). Relative expression levels were calculated using the Rotorgene Software version 1.7, which also calculates PCR efficiency. Two independent qPCR reactions were performed from two independently extracted RNA samples. Relative mRNA levels (sample of interest and U937 RNA sample) were normalized to three different housekeeping genes: the TATA-box binding protein (TBP), the ribosomal protein RPL13A and the hypoxanthine-guanine phosphoribosyltransferase (HPRT), using the following primer pairs: TBP: 5′-CCCATGACTCCCATGACC- and 3′ –TTTACAACCAAGATTCACTGTGG-; RPL13A: 5′ –CTGGACCGTCTCAAGGTGTT- and 3′ –GCCCCAGATAGGCAAACTT-; GAPDH: 5′: -GAAGGTGAAGGTCGGAGTC-and 3′ –GAAGATGGTGATGGGATTTC- [1], [45].

### Immunoblotting and antibodies

Western blots of cell lysates and virion pellets were probed with anti-V5 (Invitrogen, R690-25) or anti-HA (Roche, 11867423001) antibodies for detection of the Apobec proteins. For the quantification of XMRVgag or MoMLVgag levels on western blots, we used an anti-gag monoclonal antibody derived from the hybridoma cell line CRL1912; for the detection of env protein a polyclonal goat serum (gift from Carol Stocking, Heinrich Pette Institute, Germany) was used. Equal protein loading was verified by incubation of the blots with anti-actin Ab (Chemicon Cat.No.1501).

### Transient production of retrovirus vector pseudotypes and infection protocol

Replication incompetent Gag pseudotyped retroviral particles were produced by transient transfection of 293T cells. 5×10^6^ cells were seeded in a 10cm dish 12hrs prior transfection. 5µg pSF91-I-eGFP-PRE [41] or pM5-LTR-LUC, 10µg pSV-Mo-MLVgagpol [40] or pSV-XMRVgag1940 and 5µg of pHCMV-XMRVenv were transfected using the CaPO_4_-HBS technique according to manufacturer's instructions (Profection mammalian transfection system, Promega CatNo. E1200). 6hrs after transfection medium was replaced with 6ml DMEM/FCS containing 20mM HEPES. Supernatant was collected after 48hrs, passaged through a 0.2µm pore size filter, aliquoted and frozen at −80C.

For western blot analysis, pseudotype or replication competent supernatants were filtered and centrifuged at 110.00×g 1h at 4°C (Beckman SW60Ti). The pellet of 1ml supernatant was resuspended in 10µl PBS and analysed by immunoblotting.

### Luciferase assay

XMRV pseudotyped particles (730ng LTR-Luc reporter) were generated in the presence of transiently expressed Apobec proteins (1000ng, 500ng, 125ng, 75ng and 25ng) in a 35mm dish. Supernatants were collected as described before [10]. Briefly, 200µl viral supernatant was used to infect 5×10^4^ TE671 cells. 48h post infection cells were lysed, and luciferase activity was measured with the Infinite M200 microplate reader (Tecan) after incubating 20µl of the cell lysate with 100µl Luciferase assay substrate (Promega, E1500).

## Results

### mA3 protein as well as hA3B, hA3G and hA3F are efficiently packaged in XMRV virions

**Figure 1:**
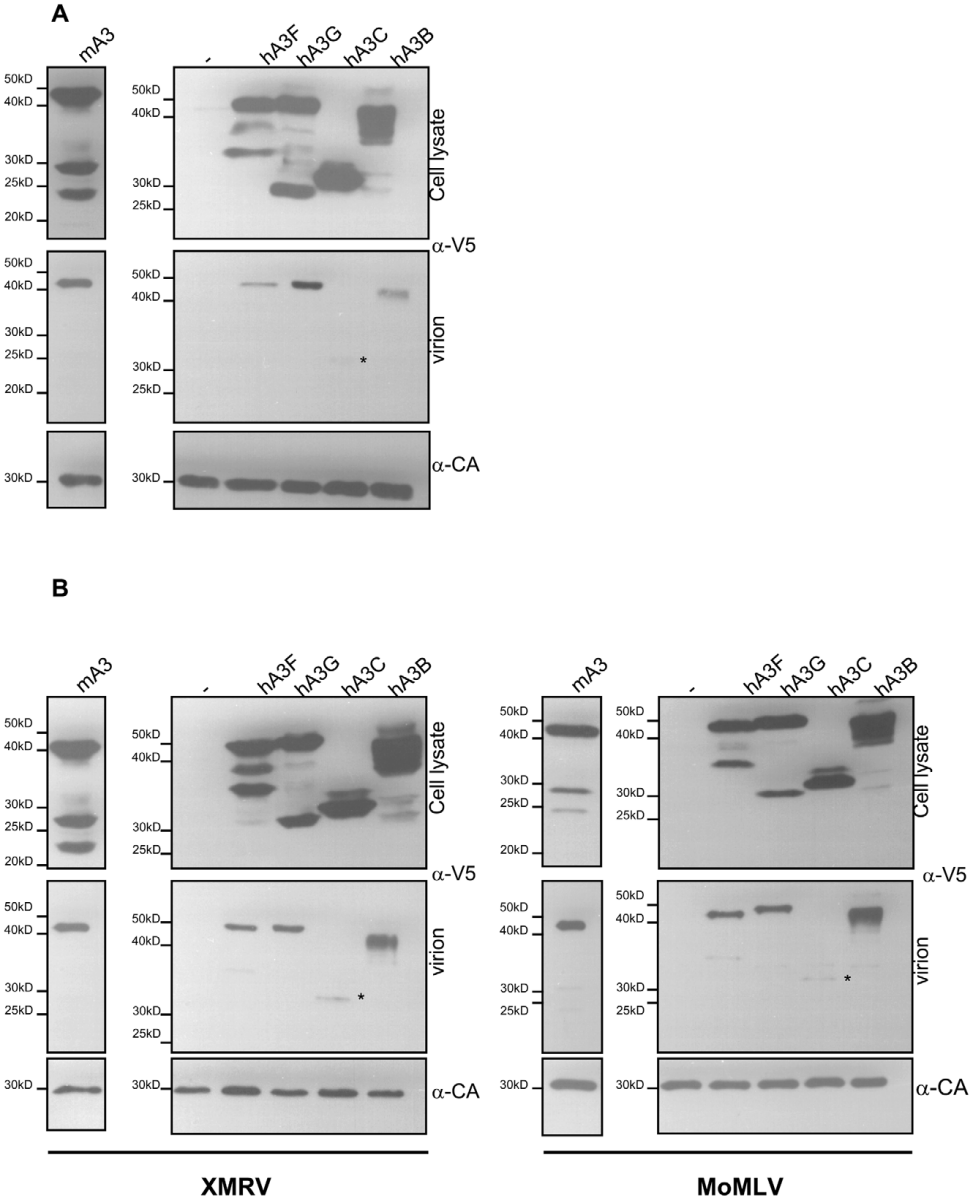
Incorporation of human Apobec 3 and mouse Apobec 3 proteins into virions. (A) Incorporation of hA3 and mA3 into XMRV virions. Virus particles were produced by transiently transfecting chronically XMRV infected 293T cells (293TX) with 150ng hA3B, hA3C, hA3F, hA3G or mA3 or control plasmid(-). Virus particles were assayed for Apobec 3 protein incorporation by immunoblotting of virus-containing pellets. Anti-HA Ab was used to detect mA3 protein and anti-V5 Ab to visualize human Apobec proteins. Loading of equal amounts of virus particles was verified by probing with a CA (p30) specific antibody (lower panel). Additionally, cell lysates of transiently transfected 293T cells were analyzed for successful Apobec protein expression (upper panel). (B) Incorporation of hA3 and mA3 protein in XMRVGag and MoMLVGag pseudotyped virus particles. 293T cells were transiently transfected with the following plasmids: XMRVgag (left panels) or MoMLVgag (right panels), LTR-Luc and pHCMV-VP62 env plasmids in the presence of 150ng hA3B, hA3C, hA3F, hA3G or mA3 or control plasmid(-).

Previously, contradictory findings about the mechanism of Apobec 3 restriction in MLV infection, in particular with regard to virion exclusion as the basis of gammaretroviruses Apobec 3 escape, have been reported [23], [35], [37]. We therefore examined packaging of mA3 protein and the human Apobec proteins hA3B, hA3C, hA3F and hA3G into XMRV virions. 293T cells chronically infected with XMRV (293TX) were transiently transfected with different Apobec 3 expression plasmids. All Apobec 3 proteins included in this study were expressed at similar levels in 293TX cell lysates 48hrs post transfection (Figure 1A). Supernatants containing XMRV virions were ultracentrifuged and incorporation of the hA3B, hA3C, hA3F, hA3G as well as the murine A3 into XMRV virions was investigated by immunoblotting. Compared to hA3F and hA3B, mA3 and hA3G exhibited much higher protein levels in the virion-associated fraction relative to the total protein amounts in the cell lysate. Surprisingly, the hA3C protein, although highly expressed in 293TX cells, was only marginally detected within XMRV virions (Figure 1).

Previously, it was reported that mA3 is cleaved by the viral protease inside the MLV particles, a putative mechanism explaining the escape of MLV from mA3 restriction [38]. We did not observe proteolysis of any of the Apobec 3 proteins incorporated into XMRV particles (Figure 1A), even after longer exposures (data not shown).

### Development of a gag-pseudotyped single round infection assay to study XMRV restriction

The gagNC region has been suggested as a domain that is responsible for efficient packaging of the Apobec proteins into virions. To develop a single round infection assay with increased virion titers and efficient packaging of the reporter construct, we replaced the gag region within the gag-pro-pol open reading frame of pSV-Mo-MLVgagpol with the XMRV gag region corresponding to nt 604 to nt 2544 (GI: 88765817) (pSV-XMRVgag1940). Each gag- encoding plasmid was transfected together with LTR-*luc* and pHCMV-XMRVenv into 293T cells in the presence or absence of transiently expressed Apobec proteins. Virion-containing supernatants were harvested 48hrs after transfection, filtered, ultracentrifuged and Apobec protein in viral particles was detected by immunoblotting (Figure 1B). For all Apobec proteins analyzed, we observed similar packaging efficacy between the XMRVgag pseudotyped virions and XMRV replication competent virion particles (Figure 1A and Figure 1B, left panel).

XMRVgag pseudotyped virions were subsequently compared to MoMLVgag pseudotyped virions with regard to Apobec packaging, as well as proteolytic cleavage of the Apobec proteins within the virion. Figure 1B demonstrates that no significant differences in incorporation of the human Apobecs B, C, F, or G as well as mouse Apobec 3 between these two gammaretroviruses were found. In accordance with previous studies [27], [29], mA3 as well as hA3G can be efficiently incorporated into MLV particles. Likewise, hA3B and hA3F are packaged by both viruses with similar efficiency. However, although hA3C is strongly expressed, we detected hardly any incorporation of the protein into either XMRV or MoMLV particles. This is in contrast to earlier reports showing efficient packaging of hA3C into MLV virions [46].

### Inhibition of XMRV infection by human A3 proteins

**Figure 2:**
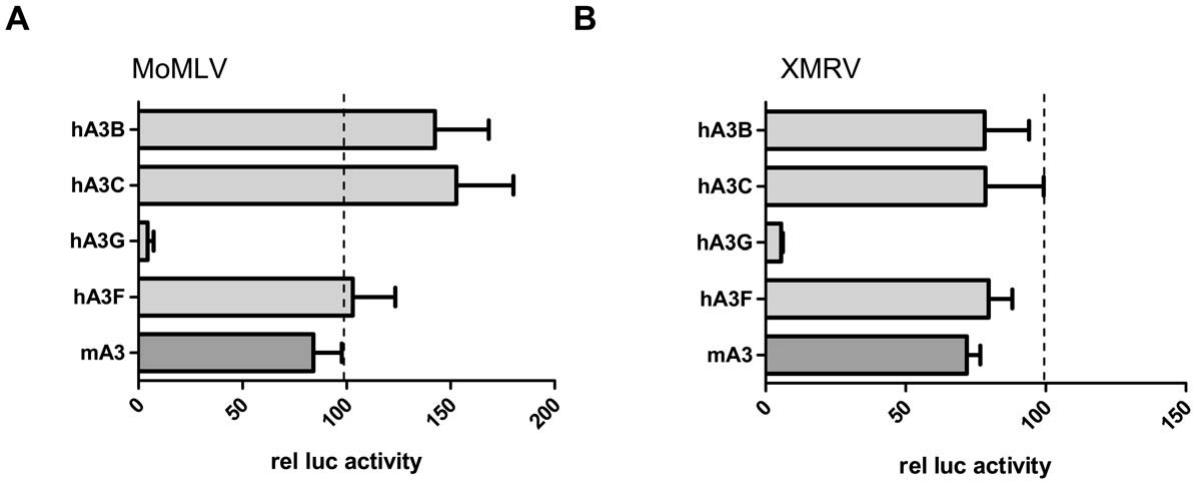
hA3G is a potent inhibitor of XMRV and MoMLV infection. 293T cells were transiently transfected with viral plasmids, a LTR-luc plasmid in the presence of 150ng Apobec or control plasmid. At 48hrs posttransfection viral supernatants were harvested, filtered and added to 293T cells. 48hrs past infection cells were lysed and luciferase activity was quantified. The level of MoMLV (left graph) or XMRV (right graph) infectivity observed in the presence of empty expression plasmid was set to 100. The indicated data represent three independent experiments performed in triplicate.

hA3G has been shown to be a potent suppressor of MLV infection in several studies [11], [27], [29], [35], [38], [47]. To examine the effect of human A3 proteins on XMRV and MoMLV infection, we tested several human Apobec 3 proteins and the mouse Apobec protein in our single round infection assay, using a constant amount of 150ng expression plasmid delivered by transient transfection. The expression of hA3G significantly (≥90%) reduced the infection of XMRVgag pseudotyped virions (Figure 2). All other tested Apobec proteins showed no statistically significant impact on the relative infection efficiency, as judged by determination of the relative luciferase activity using the empty APOBEC expression vector as control. Similar results were observed using MoMLVgag pseudotyped virions. These findings (≥95% reduction) are comparable to previous studies showing highly efficient inhibition of MLV infection by hA3G, whereas mA3G expression only marginally reduces MoMLV infectivity [27], [29], [47].

Contradictory observations have been made with regard to human Apobec 3B: while restriction of MoMLV infection has been observed by some groups [35], others did not see any effect on infection or only minor reduction of MoMLV infectivity [47]. We were unable to detect any MoMLV restriction by the h3B protein (Figure 2A); in the case of XMRV infection, a minor reduction could be observed, although the magnitude of the effect was statistically not significant when hA3B or hA3F was coexpressed (Figure 2B).

### Differences between mA3 dependent restriction of XMRV and MoMLV infectivity

**Figure 3:**
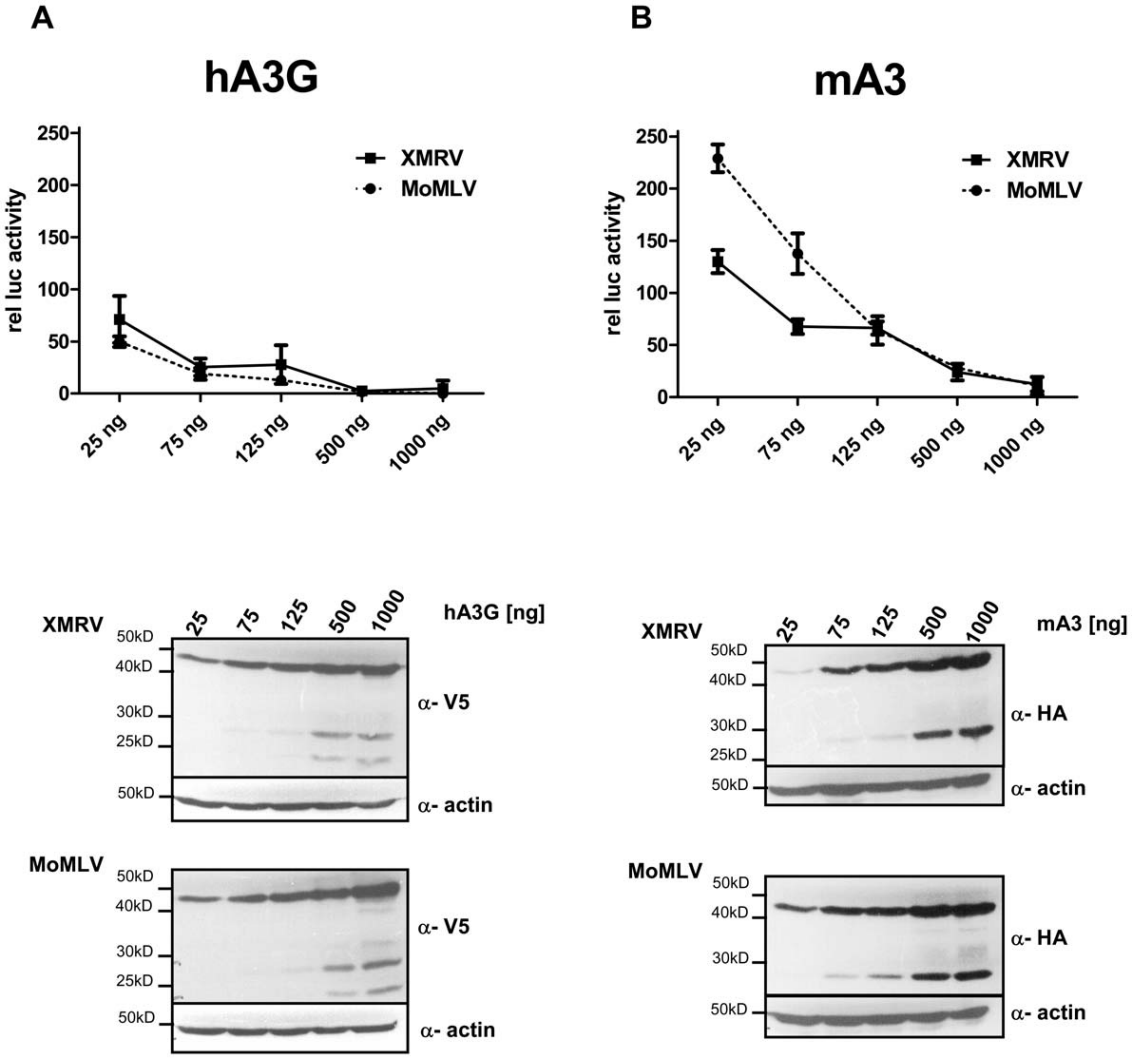
Sensitivity of XMRV and MoMLV to mA3 and hA3G restriction. LTR-Luc containing XMRV or MoMLV gag pseudotyped particles were produced in 293T cells in the presence of increasing amounts (25ng, 75ng, 150ng, 500ng, 1µg) of hA3G (A) or mA3 (B) expression plasmids, or equivalent amounts of an empty vector control (observed infectivity was set to 100). Virus titers were determined 48hrs after infection of indicator cells with 200µl viral supernatant by measuring luciferase activity. Whole cell lysates were immunoblotted to ensure increasing amounts of Apobec proteins transfected using anti V5 (hA3G) or anti-HA (mA3) antibodies. Protein loading was controlled by anti-actin staining.

We consistently detected small but reproducible differences between XMRV and MoMLV restriction by mA3. On average, the reduction of XMRV infectivity by mA3 expression was approximately 10% more efficient that that seen for MoMLV (Figure 2). To investigate the effects of mA3 and hA3G on XMRV as well as MoMLV infection in more detail, we transiently transfected 293T cells in the presence of MoMLVgag or XMRVgag together with LTR-*luc*, pHCMV-XMRVenv and increasing amounts of Apobec expression plasmids in the range from 25ng to 1µg. Supernatants were harvested 48hrs post-transfection and indicator cells (TE671 cells) were incubated with different amounts of viral supernatant. Luciferase activity was determined at 48hrs post-.infection. As expected, we observed that hA3G was a potent inhibitor of XMRV and MoMLV infection. In both cases, amounts as low as 25ng of cotransfected hA3G plasmid significantly reduced infectivity to approx. 50–60% and 125ng of the plasmid further reduced viral infectivity to ∼10–40% (Figure 3A). In comparison, mouse A3 protein is a relatively weak inhibitor of XMRV and MoMLV infection, and expression of 125ng mA3 reduced XMRV infectivity to only 70% (Figure 3B). Interestingly, in accord with our previous results at low levels of the cotransfected plasmid XMRV was consistently more sensitive to mA3 restriction when compared to MoMLV. 75ng of the mA3 expression plasmid reproducibly resulted in a observed reduction of XMRV infection to approximately 75%, whereas no significant effect on MoMLV infectivity was observed.

#### Human APOBEC 3G expression patterns in different established human cell lines and primary cells

**Figure 4:**
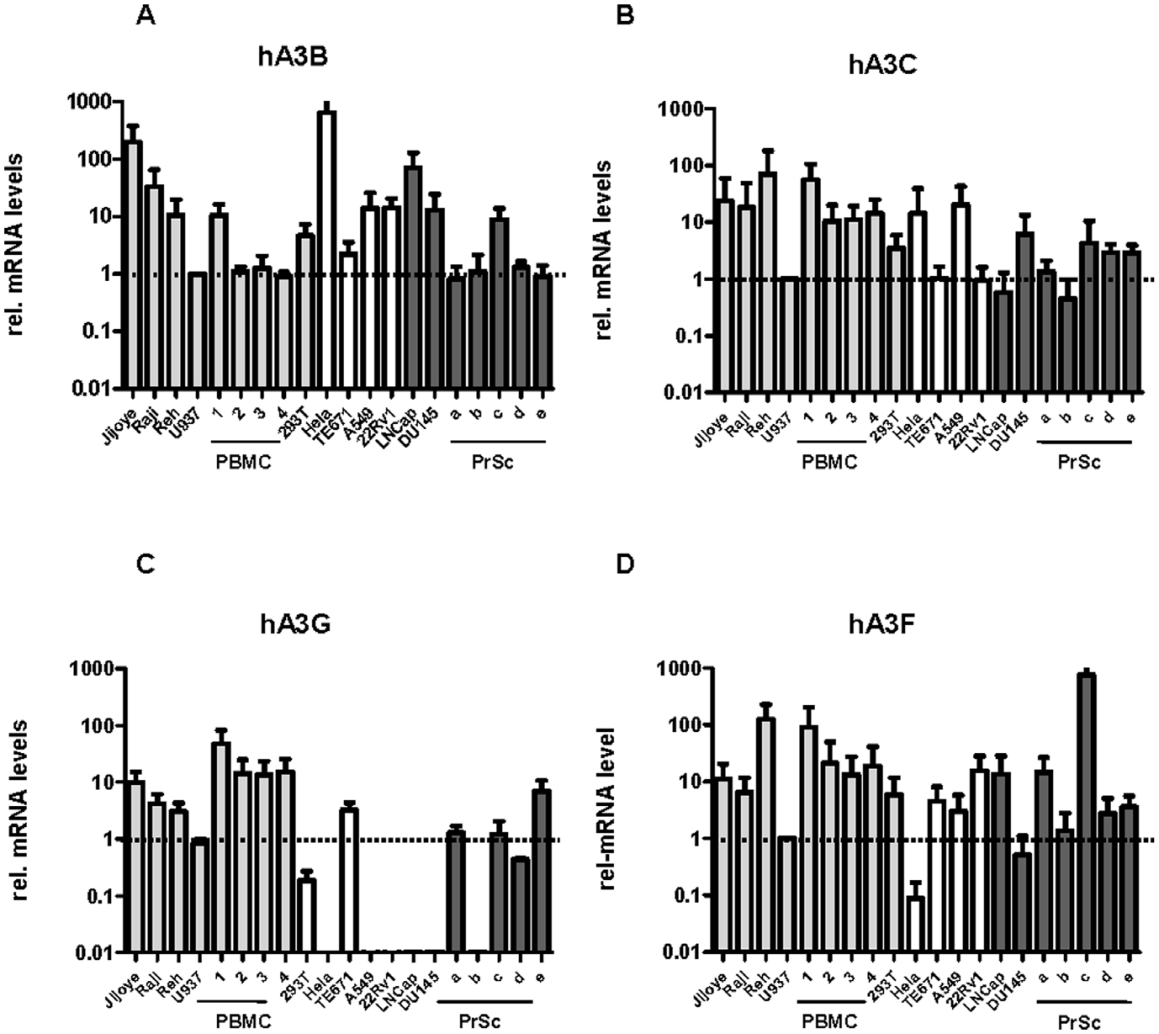
mRNA expression of hA3B, hA3C, hA3G and hA3F in human cell lines, PBMCs and primary prostatic stromal fibroblasts. (A) Relative mRNA expression levels of hA3B, (B) hA3C, (D) hA3G and (E) hA3F. Results are plotted as the mean of three different experiments relative to mRNA expression levels in U937 cells and normalized against three different housekeeping genes. Hematopoietic cell lines as well as PBMCs from different donors are indicated as light gray bars, prostate cancer cell lines and primary prostatic stromal fibroblasts (PrSc) are represented as dark grey bars and other human cell lines are shown as white bars.

In vivo, XMRV infection in familial cases of prostate cancer has been originally found to be confined to a low percentage of the stromal fibroblasts within the tumor tissue [1]. A more recent study using immunohistochemistry additionally detected viral protein expression within malignant epithelial cells of the prostate, but only rarely in stromal fibroblasts [2]. Furthermore, XMRV was recently reported to be present in PBMCs of 67% of CFS patients as well as up to 4% of healthy controls [3]. In vitro, we and others previously demonstrated that XMRV displays a xenotropic host range in vitro and efficiently infects established human cell lines as well as primary cells independent of their tissue origin [10], [12], [48]. While the XMRV promotor is more active in cells from the prostate compartment [10], [12], in vivo restriction of XMRV infection can not solely be explained by LTR-activity. We hence suspected that known retroviral restriction factors most likely play a major role in primary infection and viral spread. To investigate this hypothesis, we used quantitative RT-PCR to determine mRNA expression levels of hA3B, 3C, 3F and 3G in established human cell lines (hematopoietic cell lines: Raji, Jijoye, Reh, U937; prostate cancer cell lines 22Rv1, Du145 and LNCaP and other human cell lines: HeLa, TE671, 293T and A549 cells) (Figure 4). Relative mRNA expression levels in the various cell lines were determined by comparing Apobec 3 mRNA expression to U937 cells. Most of the tested established cell lines showed moderate to high expression levels of hA3B, 3C and 3F, whereas hA3G mRNA expression levels were more variable. All hematopoietic cell lines as well as the TE671 cells express moderate to high levels of hA3G. In contrast, hA3G was absent or expressed only at very low levels in 293T, HeLa, A549 and all of the tested prostate cancer cell lines.

In addition to established cell lines, we tested primary prostate stromal fibroblasts from different donors as well as PBMCs from different patients for their hA3G mRNA expression levels (Figure 4). In accord with the observations made with cell lines of hematopoietic origin, PBMCs showed the expected moderate to high hA3G expression [45], [49] levels. However, stromal fibroblasts isolated from prostate cancer tissue of different donors differed in their hA3G expression ranging from undetectable to moderate hA3G expression levels. Taken together, our results suggest that prostate epithelial cells or populations of hA3G-negative prostate stromal fibroblasts could be an important reservoir of XMRV infection in vivo.

## Discussion

XMRV is the first MLV-related gammaretrovirus identified in human infections [1]. In vivo, current evidence suggests that XMRV infection is restricted to specific compartments: 1–1.5% of stromal fibroblasts in the prostate have been identified to carry XMRV [1], focal infections of prostate epithelium cells in prostate cancer patients have been described [2] and recently virus transmission from activated PBMCs of CFS patients to an indicator cell line has been reported [3]. However, in vitro XMRV displays an extended cell tropism and cells from different tissue origin as well as from different species can be efficiently infected. Previously, we demonstrated that the receptor of XMRV, Xpr1 is widely expressed and although LTR-activity is dependent on the cell type [10], [12], it can not fully account for the observed in vivo restriction.

One mechanism which controls species restriction and tissue tropism of retroviruses are retroviral restriction factors, a family of proteins which form part of the host's innate immune defense repertoire. Members of the APOBEC family of cytidine deaminases are potent inhibitors of HIV1 infection as well as other retroviruses. Not surprisingly, retroviruses on the other side have evolved strategies to evade cellular restriction factors, such as post-transcriptional regulation of hA3G levels by the HIV-encoded vif protein. Since simple retroviruses (e.g. MLV, MMTV, FrMLV and XMRV) do not encode for vif homologues, the ant-restriction strategies employed by these viruses have been the subject of several studies in the past. To date, there is considerable controversy about the mechanisms by which MLV evades Apobec restriction. Virion exclusion of the Apobec proteins has been proposed as one possible mechanism [23], [35], [37], proteolytic cleavage of the virion incorporated Apobec proteins was suggested as another [38], whereas a recent publications come to the conclusion that neither mechanism is likely to account for the evasion [29].

Here, we have analyzed the effect of different human A3 proteins as well as mouse A3 on XMRV infection in a direct comparison to the closely related MoMLV. We observed that both XMRV and MoMLV can efficiently package hA3B, hA3F, hA3G and mA3. In contrast, hA3C, although efficiently expressed, can hardly be detected within virion preparations. Of all Apobec protein members which are found in the virion only hA3G strongly reduces XMRV or MoMLV infectivity; mA3 only partially reduces infectivity also at higher Apobec protein concentrations. Our findings are in agreement with recent reports demonstrating efficient packaging of both hA3G and mA3 protein into MoMLV virions, although only hA3G displays significant restriction against MoMLV infection. These results suggest that virion exclusion is not the mechanism by which MoMLV evades Apobec restriction. Additionally, we do not observe any proteolytic cleavage of mA3 within the virions of MoMLV or XMRV, although cleavage has been recently reported for MoMLV by others [27]. Recently, sensitivity of XMRV against the human retroviral restriction factors hA3G [11], [39] and tetherin was reported, whereas human TRIM5α was found to have no effect on infectivity. Additionally, the non-human factors Fv1, mA3 and primate tetherin also decreased XMRV infectivity to different extend [11]. Our results are in overall good agreement with the findings of two recent reports [11] in terms of hA3G being a potent inhibitor of XMRV infection. All reports unequivocally identify XMRV replication being highly sensitive to hA3G, whereas mA3 only partially reduces XMRV infection. Furthermore, all studies observe efficient packaging of mA3 protein into XMRV virions, and thus virion exclusion is unlikely to be the mechanism by which XMRV evades mA3 restriction. Differences observed between the current study and the recently published studies with regard to sensitivity against hA3B and hA3F are most likely due to different amounts of transiently expressed Apobec proteins used in the infectivity assays.

Interestingly, we observed differences between XMRV and MoMLV with regard to mA3 restriction sensitivity (Figure 3). Titration of the amount of transiently transfected mA3 expression levels revealed, that lower mA3 concentrations partially reduce XMRV infectivity but do not interfere with MoMLV infection. The increased sensitivity of XMRV against mA3 may suggest that, although XMRV originally originated from the mouse, it is circulating already for an evolutionary significant period of time in the human population. Thus, our findings would argue that, at least for the XMRV isolate investigated here, humans represent the current reservoir of infection.

What are the in vivo consequences of the fact that hA3G is a potent inhibitor of XMRV? So far, XMRV has been described to be present in PBMCs (67% of CFS patients as 4% of healthy controls) as well as in cells of prostate cancer tissue [1]–[3]. hA3G is widely expressed in hematopoietic cells (T-cells, B-cells and myeloid cells) and tissues with high prevalence of B- and T-cells [49]. Likewise, we detect moderate to high hA3G mRNA levels in hematopoietic cell lines as well as PBMCs. Hence, XMRV is likely to have evolved additional strategies to evade hA3G restriction in the hematopoietic compartment, which may not be detectable by the experimental in vitro procedures employed by us and others.

Interestingly, hA3G mRNA expression can not be observed in the prostate cancer cell line 22Rv1 (which has multiple copies of integrated XMRV genomes and sheds significant amount of infectious XMRV virions), LNCaP (a line which supports XMRV replication to produce high titers when infected in vitro) or DU145 cells. These findings are in line with the recent observation of Paprotka and coworkers [39], that XMRV genomes recovered from prostate cancer cell lines are hypermutated only at low frequency. In contrast, primary prostatic stromal fibroblasts isolated from different donors (patients undergoing prostatectomy) displayed variable levels of hA3G expression, ranging from being undetectable to moderate. Prostate epithelial cells or a population of prostatic stromal fibroblasts with low A3G expression could be one important reservoir for XMRV replication in vivo.

## Supporting Information

Supplementary Figure S1 qPCR amplification ranges and efficiencies of each individual primer set used in the study.
(0.23 MB TIF)
