# Supplementary figures and images for: Apobec 3G Efficiently Reduces Infectivity of the Human Exogenous Gammaretrovirus XMRV
Source: PLoS One. 2010 Jul 23;5(7):e11738. doi: 10.1371/journal.pone.0011738 (PMC2909211; doi:10.1371/journal.pone.0011738)

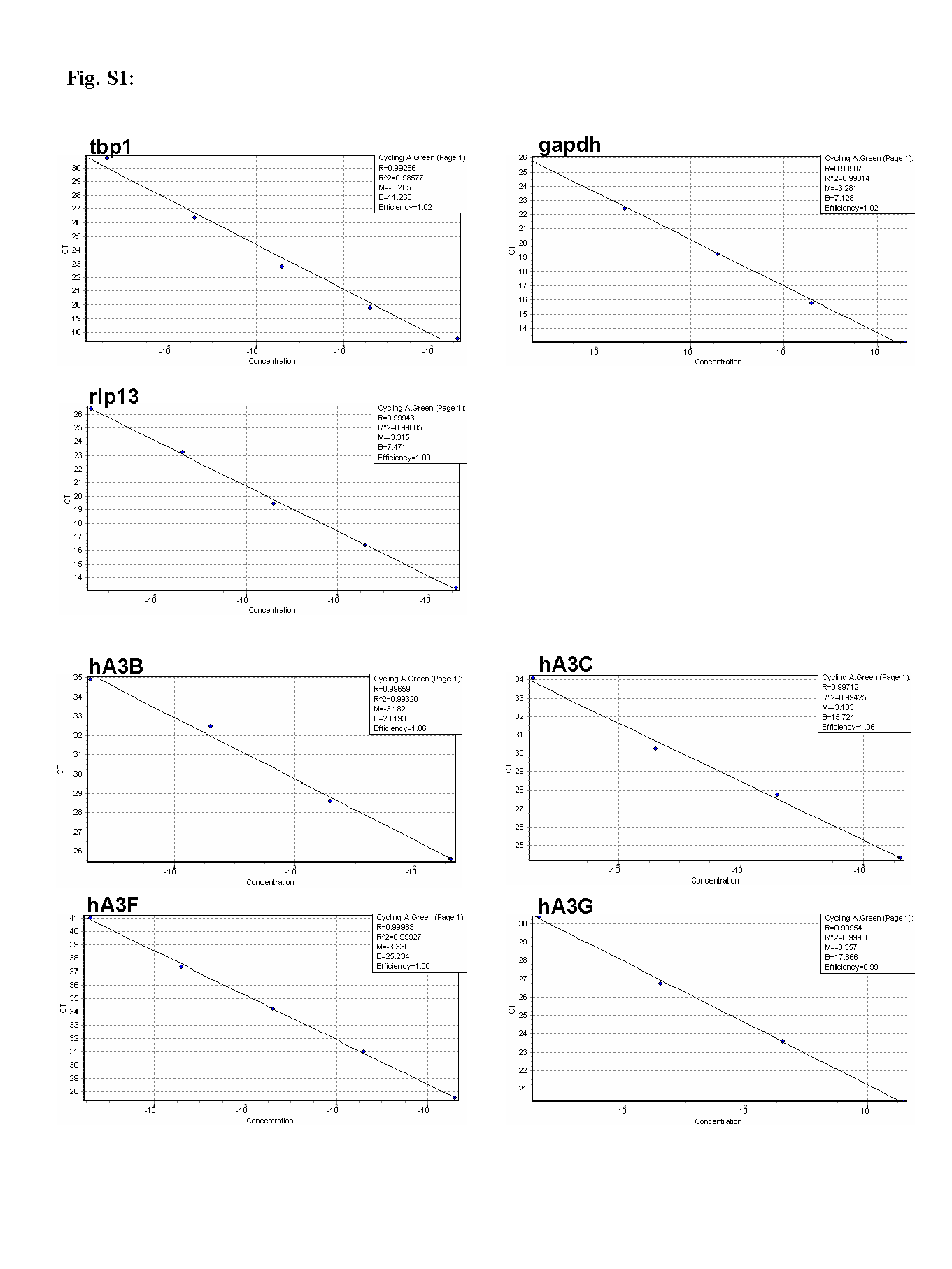

Supplement: Supplementary Figure S1 — qPCR amplification ranges and efficiencies of each individual primer set used in the study. (0.23 MB TIF) [file pone.0011738.s001.tif]
